# 797 Single Stage Application of Autologous Skin Cell Suspension in Deep Partial Thickness Pediatric Facial Burns

**DOI:** 10.1093/jbcr/irac012.347

**Published:** 2022-03-23

**Authors:** Richard B Lou, Anjay Khandelwal, Beverly Beaucock

**Affiliations:** Akron Children's Hospital, Akron, Ohio; Akron Children's Hospital, Akron, Ohio; Akron Children's Hospital, Akron, Ohio

## Abstract

**Introduction:**

Deep partial thickness facial burns in the pediatric population present a dilemma. Standard burn treatment includes excision and split thickness sheet grafting for the face or closure which often yield sub-optimal results. Point-of-care system for autologous skin cell suspension (ASCS) can be utilized as a stand alone option for partial thickness burns. We sought to evaluate our early results from the treatment of pediatric deep partial thickness facial burns using ASCS.

**Methods:**

This was a retrospective cohort analysis of pediatric deep partial thickness facial burns treated with early excision and application of ASCS. After assessment by two surgeons, patients underwent tangential excision with either standard surgical burn knives or dermabrasion. Depth was assessed and if dermal elements were present, then ASCS was applied. Primary outcome was time to >95% reepithelialization, and secondary outcomes were aesthetics, Vancouver Scar Scale, postoperative complications, number of reoperations for wound closure, and need for burn reconstruction.

**Results:**

From September 2020 to July 2021, eight pediatric patients (six male and two female) with deep partial thickness facial burns were treated within one week of injury with excision and ASCS. TBSA ranged from 0.3%-24% with some having additional anatomic areas. The mean age was 2.5 years old, with a range of 1.08-5.0 years old. Burn mechanisms included flame, scald, and contact. The mean time from burn injury to initial excision and ASCS was 4.3 days, with a range of 2-7 days. Average time to complete reepithelialization was 20.3 days, with a range of 4-47 days. Average follow up duration was 218 days with a range of 98-365 days. No patients experienced complete graft loss or infection. One patient required need for reoperation with reapplication of polylactic acid skin substitute placed to a small area on the face during closure for other non-face burns. Of the 8 patients, the two patients with the longest times to complete reepithelialization required laser therapy, of which one patient also needed limited facial scar contracture release.

**Conclusions:**

Early, single-staged ASCS application results in good aesthetic and relatively quick healing times with no complications and limited need for additional burn reconstruction. While the results are promising, there is a need for larger studies with longer term follow up before wider applicability.

